# A loss-of-function and H2B-Venus transcriptional reporter allele for *Gata6* in mice

**DOI:** 10.1186/s12861-015-0086-5

**Published:** 2015-10-24

**Authors:** Laina Freyer, Christian Schröter, Néstor Saiz, Nadine Schrode, Sonja Nowotschin, Alfonso Martinez-Arias, Anna-Katerina Hadjantonakis

**Affiliations:** Developmental Biology Program, Sloan Kettering Institute, Memorial Sloan Kettering Cancer Center, New York, NY 10065 USA; Department of Genetics, University of Cambridge, Cambridge, CB2 3EH UK

**Keywords:** GATA6, H2B-Venus, Reporter mice, Endoderm, Cardiac mesoderm, Live Imaging

## Abstract

**Background:**

The GATA-binding factor 6 *(Gata6)* gene encodes a zinc finger transcription factor that often functions as a key regulator of lineage specification during development. It is the earliest known marker of the primitive endoderm lineage in the mammalian blastocyst. During gastrulation, GATA6 is expressed in early cardiac mesoderm and definitive endoderm progenitors, and is necessary for development of specific mesoderm and endoderm-derived organs including the heart, liver, and pancreas. Furthermore, reactivation or silencing of the *Gata6* locus has been associated with certain types of cancer affecting endodermal organs.

**Results:**

We have generated a *Gata6*^*H2B-Venus*^ knock-in reporter mouse allele for the purpose of labeling GATA6-expressing cells with a bright nuclear-localized fluorescent marker that is suitable for live imaging at single-cell resolution.

**Conclusions:**

Expression of the Venus reporter was characterized starting from embryonic stem (ES) cells, through mouse embryos and adult animals. The Venus reporter was not expressed in ES cells, but was activated upon endoderm differentiation. *Gata6*^*H2B-Venus/H2B-Venus*^ homozygous embryos did not express GATA6 protein and failed to specify the primitive endoderm in the blastocyst. However, null blastocysts continued to express high levels of Venus in the absence of GATA6 protein, suggesting that early *Gata6* transcription is independent of GATA6 protein expression. At early post-implantation stages of embryonic development, there was a strong correlation of Venus with endogenous GATA6 protein in endoderm and mesoderm progenitors, then later in the heart, midgut, and hindgut. However, there were discrepancies in reporter versus endogenous protein expression in certain cells, such as the body wall and endocardium. During organogenesis, detection of Venus in specific organs recapitulated known sites of endogenous GATA6 expression, such as in the lung bud epithelium, liver, pancreas, gall bladder, stomach epithelium, and vascular endothelium. In adults, Venus was observed in the lungs, pancreas, liver, gall bladder, ovaries, uterus, bladder, skin, adrenal glands, small intestine and corpus region of the stomach. Overall, Venus fluorescent protein under regulatory control of the *Gata6* locus was expressed at levels that were easily visualized directly and could endure live and time-lapse imaging techniques. Venus is co-expressed with endogenous GATA6 throughout development to adulthood, and should provide an invaluable tool for examining the status of the *Gata6* locus during development, as well as its silencing or reactivation in cancer or other disease states.

**Electronic supplementary material:**

The online version of this article (doi:10.1186/s12861-015-0086-5) contains supplementary material, which is available to authorized users.

## Background

GATA-binding factor 6 (GATA6) is a member of the GATA family of zinc finger transcription factors that are characterized by their DNA binding domain [1]. GATA factors are highly conserved across vertebrate species, which include six members of the family [2, 3]. They are also evolutionarily conserved among invertebrates (*D. melanogaster* and *C. elegans*) where they participate in heart and endoderm formation [4]. In humans, *de novo* mutations in the *Gata6* locus cause haploinsufficiency that is associated with congenital heart malformations and neonatal diabetes due to pancreatic agenesis [5–10].

During pre-implantation embryo development in mice, *Gata6* is required for formation of extra-embryonic tissues [11–14]. In the early mouse blastocyst (32–64 cell stage), GATA6 protein is uniformly expressed in the inner cell mass (ICM) and trophectoderm (TE) [12, 15]. By the mid-blastocyst (64–100 cell) stage, expression of GATA6 in the ICM becomes restricted in a mosaic ‘salt-and-pepper’ pattern [15, 16] and co-localizes with GATA4 [15, 17]. At this stage, exclusive enrichment of GATA6 serves as the earliest known determinant of the primitive endoderm (PrE) lineage, which is the precursor to the parietal endoderm (ParE) and visceral endoderm (VE) [18–20]. GATA6 is necessary for PrE specification in the mouse embryo, and either GATA6 or GATA4 are sufficient to promote differentiation into extra-embryonic endoderm from embryonic stem (ES) cells [13, 14, 21, 22]. *Gata6*^*+/−*^ heterozygotes have delayed PrE specification and a reduction in the number of cells that adopt a PrE fate at the late blastocyst stage [13, 14].

Following implantation, strong *Gata6* expression continues in extra-embryonic tissues; namely the ParE which deposits Reichert’s membrane that lines the parietal yolk sac, and the allantois which will contribute blood vessels to the umbilical cord [18]. Weaker expression of *Gata6* mRNA can be observed in the VE that gives rise to the visceral yolk sac and a fraction of the embryonic gut endoderm, although GATA6 protein levels in the VE are diminished by gastrulation stages [18, 19, 23, 24]. Tetraploid embryo complementation, where only the embryonic tissue was null for *Gata6*, demonstrated that GATA6 is indispensible for embryonic liver development [25]. Heart development in contrast does not require embryonic expression of *Gata6*, likely due to compensation by *Gata4*, which shares 90 % amino acid sequence homology with the DNA-binding domain of GATA6 [26] and is also expressed in the myocardium [25]. When both factors are conditionally deleted, cardiac progenitors are specified even though the heart does not form [27]. *Gata6*^*+/−*^*;Gata4*^*+/−*^ compound heterozygotes die by E13.5 with cardiovascular anomalies [28].

After gastrulation, *Gata6* is expressed in the cardiac crescent at the headfold stage (E7.75), as well as in the lateral plate mesoderm, primary and secondary heart fields, and heart tube [18, 27]. By E9.5, *Gata6* expression is restricted to the heart myocardium and gut endoderm where it persists throughout development [12, 18]. Later onset of *Gata6* expression during development is observed in arterial smooth muscle cells, the bladder, lung bronchi, and the urogenital ridge; none of which co-express *Gata4* [18]. However, both *Gata6* and *Gata4* are expressed throughout the pancreatic epithelium during early specification and expansion. Then, later in development their expression domains become mutually exclusive with *Gata6* restricted to cells of the endocrine pancreas [29, 30]. When either factor alone is conditionally ablated in the pancreas, only mild and non-persisting defects are observed. However, tissue-specific deletion of both GATA6 and GATA4 factors results in pancreatic agenesis [31].

In adult organs, *Gata6* expression continues in the heart, lung, stomach, small intestine, liver, bladder, pancreas, adrenal glands, ovaries, and skin [18, 32–36]. Developmental expression of GATA6 is extensive in the intestinal epithelium, but later becomes exclusive to the enteroendocrine lineage of adults [37, 38]. GATA6 is also the only GATA family member that is expressed in adult vascular smooth muscle cells [32, 39]. Misregulation of GATA6 has been linked to various tumor expression profiles. Loss of GATA6 is common in ovarian cancer and may lead to de-differentiation of ovarian epithelial cancer cells and increased occurrence of aneuploidy [40]. Reduced GATA6 activity may directly impact metastatic progression of lung adenocarcinoma [41], while overexpression of GATA6 is associated with poor prognosis in esophageal adenocarcinoma [42]. In colorectal cancer, high levels of GATA6 predict the likelihood of metastasis to the liver [43], and overexpression may promote survival of oncogenic cells in gastric cancer [44]. GATA6 is also a useful marker of pediatric germ cell tumors [35].

Given the importance of *Gata6* as a key regulatory factor during development as well as in particular adult organs, it would be useful to have a method of identifying and tracing the fate of *Gata6* expressing cells. While other *Gata6* transcriptional *LacZ* reporters exist [12, 45], a nuclear-localized fluorescent reporter instead would be suitable for live imaging and cell sorting. In this report, we describe a new *Gata6*^*H2B-Venus*^ knock-in mouse line that acts as both a loss-of-function and transcriptional reporter allele. In mice, the bright nuclear-localized H2B-Venus yellow fluorescent protein reporter correlates well with endogenous GATA6 protein and recapitulates tissue-specific expression patterns from pre-implantation stages of embryonic development to adulthood. *Gata6*^*H2B-Venus*^ will be a useful reporter for live imaging the dynamics of transcriptional activation in individual cells that are expressing, or recently have expressed *Gata6*. It could also be used for studies and analysis of *Gata6* expression in endoderm and mesoderm lineages in mice, including the isolation of these cell populations. Furthermore, expression of *Gata6*^*H2B-Venus*^ could be utilized to assess misregulation of the locus that may occur at the onset and/or as a consequence of disease states such as cancer.

## Results and discussion

### Generation of *Gata6*^*H2B-Venus*^ knock-in reporter mice

**Fig. 1:**
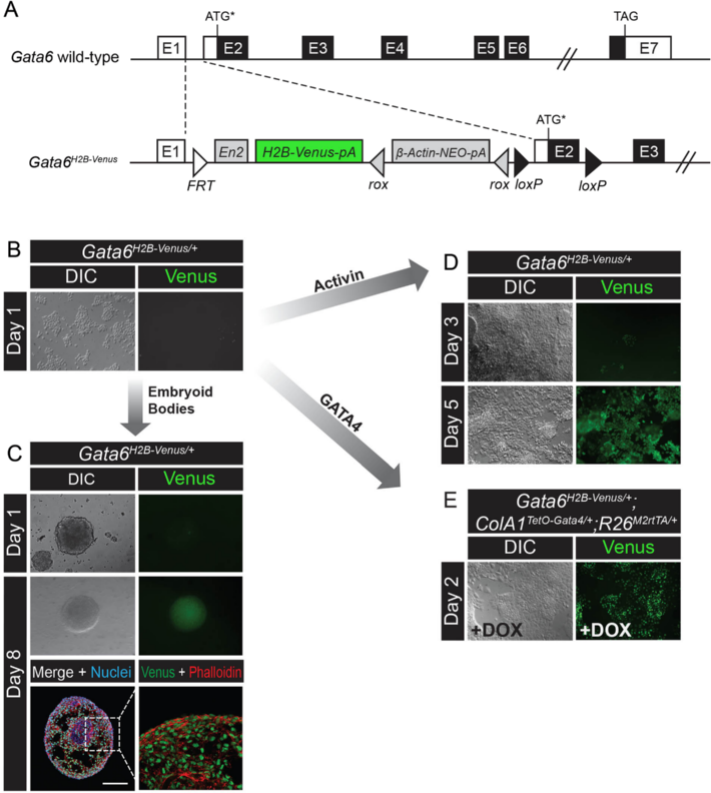
*Gata6* ^*H2B-Venus*^ targeting strategy and reporter expression during endoderm differentiation of ES cells. **a** The wild-type *Gata6* and targeted *Gata6* ^*H2B-Venus*^ alleles. Exon 1–7 (E1-7), non-coding regions (white boxes), open reading frame (black boxes), *Engrailed 2* (*En2*), Neomycin cassette (NEO), single polyadenylation sequences (pA), *rox* sites (grey triangles), *loxP* sites (black triangles), *FRT* site (white triangle), start codon (ATG), stop codon (TAG). Asterisk indicates that the ATG is one of two translational start sites located within Exon 2. **b** *Gata6* ^*H2B-Venus/+*^ ES cells do not express Venus while maintained in the pluripotent state in media containing serum and LIF. **c** Embryoid body formation from *Gata6* ^*H2B-Venus/+*^ ES cells. Expression of Venus occurs on the surface of embryoid bodies by Day 8. Cryosections stained with Phalloidin showed expression of Venus both on the surface and inside the embryoid bodies. Scale bar is 100 μm. **d** Growth factor treatment using Activin A directed *Gata6* ^*H2B-Venus/+*^ ES cell differentiation into the endoderm lineage resulting in upregulation of Venus by Day 5. **e** Overexpression of a single copy of GATA4-mCherry using the Tet-ON system in *Gata6* ^*H2B-Venus/+*^ *;ColA1* ^*TetO-Gata4-mCherry/+*^ *;R26* ^*M2rtTA/+*^ ES cells. Upon treatment with Doxycycline (DOX) for 48 h, cells were driven to differentiate towards the extraembryonic endoderm lineage resulting in activation of Venus expression. Differential interference contrast (DIC)

An enhancer element located −8 kb from the *Gata6* transcriptional start site is sufficient to drive expression of GATA6 in the heart and gut [46–48]. To obtain a reporter that recapitulates the full spectrum of *Gata6* transcriptional control, we targeted the endogenous *Gata6* locus by modification of a EUCOMM knockout-first construct [49, 50]. Specifically, we targeted H2B-Venus to the first non-coding intron of the mouse *Gata6* gene, upstream of two alternative translation initiation codons located 438 bp apart from one another within Exon 2 (E2, Fig. 1a) [48]. Targeted *Gata6*^*H2B-Venus/+*^ ES cells were injected into mouse blastocysts to generate chimeric mice.

Expression of Venus was examined in ES cells, which normally do not express GATA6. Accordingly, the Venus reporter was not detected in pluripotent *Gata6*^*H2B-Venus/+*^ ES cells (Fig. 1b). To determine if *Gata6*^*H2B-Venus/+*^ performs faithfully as a reporter in cells, we directed the differentiation of ES cells into endoderm, which expresses *Gata6*, using three different methods. First, *Gata6*^*H2B-Venus/+*^ ES cells were cultured under conditions to promote formation of embryoid bodies, which are suspended aggregates of cells capable of differentiating into all three germ lineages. Embryoid bodies did not initially express Venus, however Venus + cells were observed by Day 8 in both endoderm cells located on the surface of the bodies as well as in mesoderm progenitors located inside (Fig. 1c). Using growth factors, we also differentiated *Gata6*^*H2B-Venus/+*^ ES cells into definitive endoderm. Upon treatment with Activin A, GSK3 inhibitor, and the BMP inhibitor Dorsomorphin [51], activation of Venus was seen in *Gata6*^*H2B-Venus/+*^ cells starting at Day 3 and increasing in frequency up to Day 5 (Fig. 1d). Finally, *Gata6*^*H2B-Venus/+*^ ES cells were directed to differentiate towards a PrE fate by transiently overexpressing GATA4-mCherry, making use of a single-copy Tet-ON system for inducible gene expression formed by *ColA1*^*TetO-Gata4-mCherry/+*^*and R26*^*M2rtTA/+*^ alleles present in the background of reporter ES cells [50, 52, 53]. Upon treatment with Doxycycline for 2 days, induction of GATA4-mCherry was sufficient to activate expression of Venus in *Gata6*^*H2B-Venus/+*^*;ColA1*^*TetO-Gata4-mCherry/+*^*;R26*^*M2rtTA/+*^ ES cells (Fig. 1e, [50]).

### Expression of H2B-Venus reporter is restricted to primitive endoderm

**Fig. 2:**
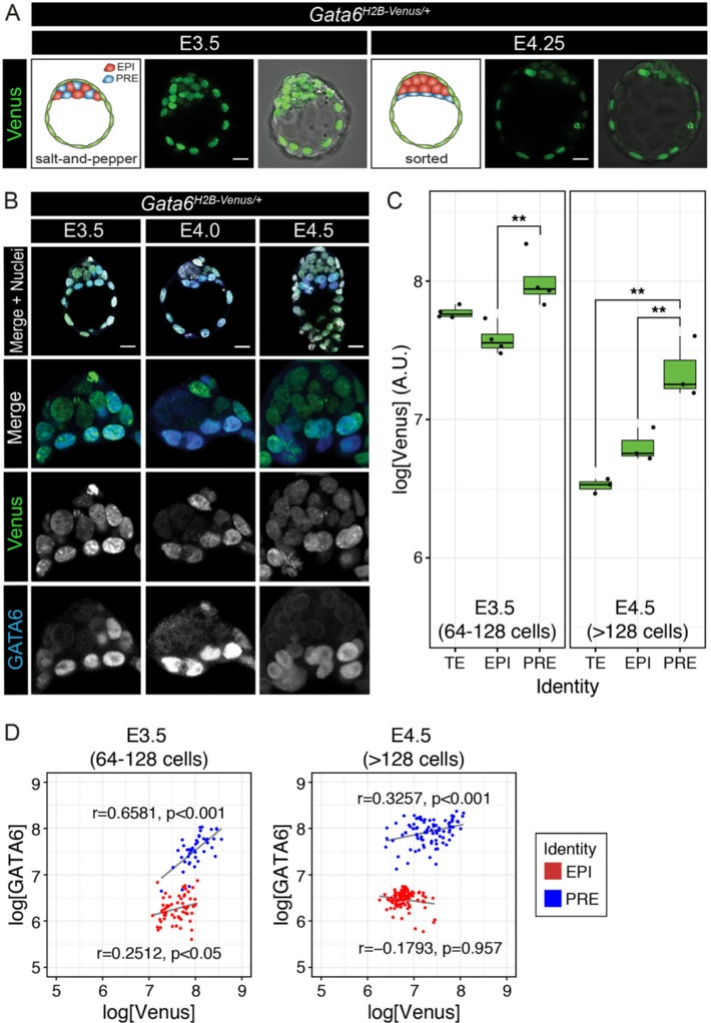
Venus and GATA6 expression in blastocyst-stage embryos. **a** Live imaging of *Gata6* ^*H2B-Venus/+*^ heterozygous blastocysts for 17 h from E3.5-E4.25. Venus is expressed both in the ICM and TE. Schematic diagram of E3.5 stage blastocyst depicts ‘salt-and-pepper’ distribution of cells fated to PrE (GATA6+, blue) and Epi (NANOG+, red), while TE is indicated as green. By E4.5, PrE and Epi cells have sorted to the exterior versus interior of the ICM, respectively. **b** Co-localization of Venus (green) with endogenous immunolabeled GATA6 protein (blue) in fixed *Gata6* ^*H2B-Venus/+*^ embryos. Nuclei are stained with Hoechst (grey). For (**a**) and (**b**), images are 5 μm projections of whole *z*-stacks. **c** Box plots showing the level of Venus expression in each blastocyst lineage (TE, Trophectoderm; EPI, Epiblast; PRE, Primitive Endoderm) in *Gata6* ^*H2B-Venus/+*^ heterozygous blastocysts at E3.5 and E4.5. Each dot represents the average level of Venus expression (as logarithm) for each embryo and lineage (*n* = 4 embryos at E3.5; *n* = 3 embryos at E4.5). PrE cells expressed significantly higher levels of Venus than EPI cells at both stages (ANOVA, with post-hoc Tukey’s range test; *p* < 0.01). **d** Scatter plots showing the expression of Venus and endogenous GATA6 (as logarithms) in ICM cells of all embryos at each stage (blue: PrE; red: EPI cells). Expression levels of Venus show a positive and significant correlation with those of GATA6 PrE cells (blue) at both E3.5 and E4.5. A weak correlation between Venus and GATA6 levels was observed in the EPI of E3.5 but not E4.5 embryos. Pearson’s correlation coefficient and p values are shown in the graphs next to the corresponding group. ** = *p* < 0.01 Scale bars = 20 μm

To assess Venus expression in live embryos, *Gata6*^*H2B-Venus/+*^ blastocysts were collected at E3.5 and imaged over the course of 17 h by laser scanning confocal microscopy. At these stages, Venus was expressed in the ICM and TE at levels that were bright enough for live time-lapse imaging (Fig. 2a). Furthermore, differential levels of nuclear Venus signal within the ICM were detected. To assess differential expression of Venus in each blastocyst lineage, *Gata6*^*H2B-Venus/+*^ blastocysts were fixed at E3.5 (*n* = 4 embryos) and E4.5 (*n* = 3 embryos) and stained for endogenous GATA6 (Fig. 2b). Overall, cells that expressed Venus also expressed GATA6, mainly in the PrE. However, Venus was present in GATA6-negative cells in the ICM, possibly due to translational repression mediated by sequences present in the wild-type *Gata6* mRNA but not the reporter mRNA, or as a consequence of the longer half-life of the H2B-Venus reporter compared to GATA6 protein. For example, Notch signaling reporter mice also express an *H2B-Venus* reporter and, in these mice, the perdurance of the reporter protein acts as a short-term lineage tracer of cells receptive to Notch signaling [54].

Perdurance of Venus may explain low-level expression that continues in the epiblast (Epi) that may be prohibitive for its use as a PrE-only reporter in the blastocyst, but could remain useful as a short-term lineage tracer. Confocal z-stacks were segmented using the nuclear segmentation algorithm MINS (Modular Interactive Nuclear Segmentation) to quantify fluorescence intensity in single cells. PrE cells (GATA6 positive) displayed significantly higher levels of Venus than Epi cells at both blastocyst stages (*p* < 0.01, Fig. 2c). Furthermore, we observed a highly significant correlation between the level of GATA6 and Venus protein levels in PrE cells at both E3.5 (*r* = 0.6581, *p* < 0.001) and E4.5 (*r* = 0.3257, *p* < 0.001; Fig. 2d). At E3.5, we also observed a weak, although significant correlation (*r* = 0.2512, *p* < 0.05) between the levels of GATA6 and Venus in Epi cells (Fig. 2d). The absence of correlation in Epi cells at E4.5 (*r* = −0.1793, *p* = 0.957; Fig. 2d) suggests that the correlation at E3.5 may be due to residual GATA6 protein found in Epi cells at that stage.

### *Gata6*^*H2B-Venus*^ is a loss-of-function allele

**Fig. 3:**
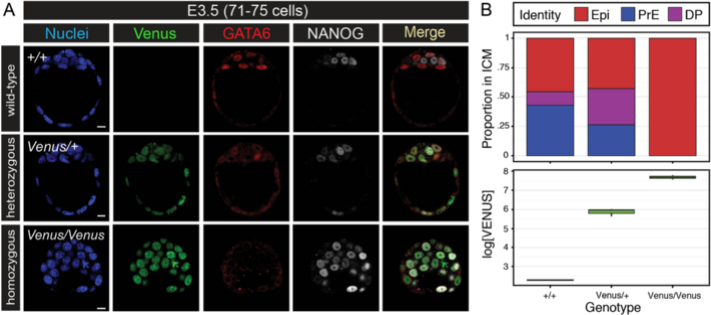
*Gata6* ^*H2B-Venus/H2B-Venus*^ homozygous blastocysts exhibit a *Gata6* null phenotype. **a** Immunofluorescence for GATA6 and NANOG protein in *Gata6* ^*+/+*^ (*+/+*; wild-type) *Gata6* ^*H2B-Venus/+*^ (*Venus/+*; heterozygous) and *Gata6* ^*H2B-Venus/H2B-Venus*^ (*Venus/Venus*; homozygous) blastocysts at E3.5. *Gata6* ^*H2B-Venus/H2B-Venus*^ mutants did not express GATA6 protein, and instead expressed NANOG in all ICM cells even though the reporter is transcriptionally active. Nuclei are stained with Hoechst (blue). **b** Quantification of cells with epiblast (EPI, NANOG+) versus PrE (PRE, GATA6+) identity at E3.5. Cells that did not adopt a clear identity were marked as double positive (DP) for both NANOG and GATA6. *Gata6* ^*H2B-Venus/H2B-Venus*^ homozygotes did not specify PrE, and *Gata6* ^*H2B-Venus/+*^ heterozygotes had a relatively reduced number of cells with PrE identity and increased numbers of double positive cells with undecided identity. *Gata6* ^*H2B-Venus/H2B-Venus*^ embryos with both alleles of the reporter, and effectively a *Gata6 *null,  had higher levels of Venus expression compared to *Gata6* ^*H2B-Venus/+*^ embryos with only one reporter allele. This would suggest that GATA6 either does not regulate, or negatively feeds back on, *Gata6* gene expression.

To confirm that *Gata6*^*H2B-Venus*^ is a loss-of-function allele for endogenous GATA6, *Gata6*^*H2B-Venus/H2B-Venus*^ blastocysts were immunostained for expression of GATA6 (labels the PrE) and NANOG (labels the Epi) at E3.5 (Fig. 3a). At this time, GATA6 and NANOG expression begin to resolve into a mutually exclusive ‘salt-and-pepper’ expression pattern defining PrE and Epi precursors respectively. This occurs prior to lineage segregation in which PrE cells are sorted to the surface while Epi cells remain in the ICM. *Gata6*^*H2B-Venus/H2B-Venus*^ homozygous blastocysts did not express GATA6 protein and instead expressed NANOG in all ICM cells which fail to specify PrE, similar to what was observed in mutants made with other *Gata6* null alleles (Fig. 3a, [13, 14]. Venus continued to be robustly expressed in the ICM in the absence of GATA6 protein, possibly due to the presence of transcriptional machinery that normally activates the *Gata6* locus despite the inability to produce GATA6 protein. Alternatively, it could mean that downstream factors that typically repress the *Gata6* locus are dependent on GATA6 protein. Again, MINS was employed on confocal z-stacks to quantify fluorescence intensity in single cells. As expected, *Gata6*^*H2B-Venus/+*^ heterozygous blastocysts had a reduced number of cells that adopted a PrE fate (Fig. 3b) [13]. The levels of Venus in *Gata6*^*H2B-Venus/H2B-Venus*^ homozygous embryos were higher compared to *Gata6*^*H2B-Venus/+*^ heterozygous embryos (Fig. 3b). This could be due to bi-allelic expression of the reporter in homozygous embryos.

### Early post-implantation expression of *Gata6*^*H2B-Venus*^

To assess the expression of *Gata6*^*H2B-Venus*^ at early post-implantation stages, *Gata6*^*H2B-Venus/+*^ embryos were collected from E5.5 to E6.0 and immunostained for endogenous GATA6 protein.

**Fig. 4:**
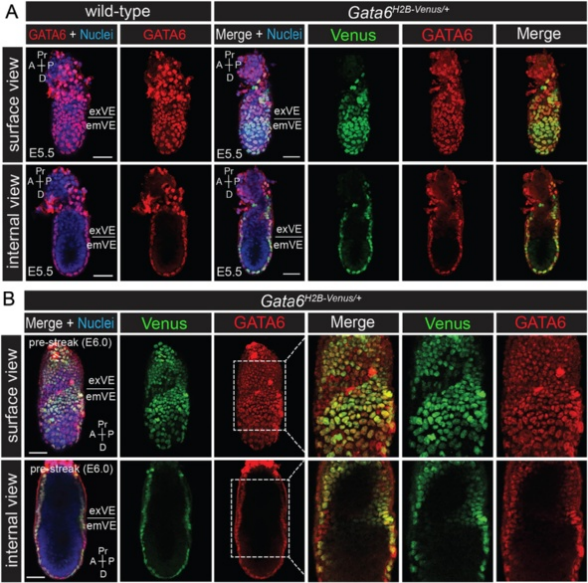
Co-expression of the *Gata6* ^*H2B-Venus*^ reporter with endogenous GATA6 during early post-implantation development. **a** Wholemount immunofluorescence for endogenous GATA6 protein (*red*) on wild-type and *Gata6* ^*H2B-Venus/+*^ embryos at E5.5. Projected surface views showed that Venus and GATA6 were mostly co-localized in the VE. Corresponding projections showing internal views of the same embryos demonstrated that neither Venus nor GATA6 were expressed in the embryonic or extra-embryonic ectoderm at these stages. **b** Wholemount immunofluorescence for endogenous GATA6 protein (*red*) on *Gata6* ^*H2B-Venus/+*^ embryos at E6.0 (pre-streak, PS). The dashed white boxes indicate regions of higher magnification showing Venus and GATA6 expression in the VE. It should be noted that red fluorescent signal in the apical VE is likely due to non-specific binding of the GATA6 antibody to the surface of the extraembyonic tissue. Surface and internal views are renderings of *z*-series images. Extra-embryonic visceral endoderm (exVE), embryonic visceral endoderm (emVE), proximal (Pr), distal (D), anterior (A), posterior (P). Nuclei were stained with Hoechst (*blue*). Scale bars are 50 μm

**Fig. 5:**
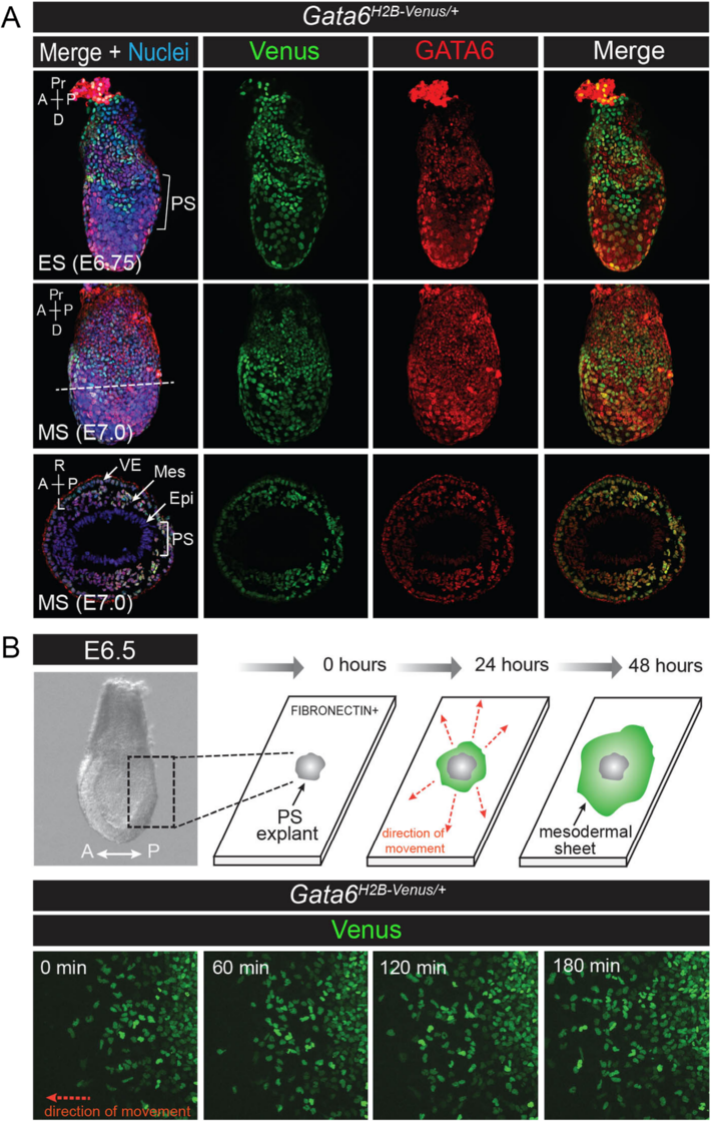
*Gata6* ^*H2B-Venus*^ reporter is expressed in nascent endoderm and mesoderm during gastrulation. **a** Wholemount immunofluorescence for endogenous GATA6 protein (*red*) on *Gata6* ^*H2B-Venus/+*^ embryos at early streak (ES, E6.75) and mid-streak (MS, E7.0). Venus is expressed in a mosaic pattern in the VE. The PS region is indicated in the posterior portion of the embryo (bracket). Transverse sections through the PS at E7.0 (level of section indicated by dotted line) are shown below. GATA6 and Venus are expressed in cells that have ingressed from the PS and are migrating laterally to the anterior of the embryo. Venus and GATA6 are not expressed in the Epi, weakly expressed in the VE, and heterogenously expressed in the intervening mesodermal wings (Mes). The red fluorescent signal in the apical VE that forms a ring around the tissue section is likely due to non-specific binding of the GATA6 antibody to the surface of the extraembyonic tissue. **b** PS explants to track movements of migrating Venus + cells *in vitro*. The PS region was dissected from the posterior portion of E6.5 *Gata6* ^*H2B-Venus/+*^ embryos and germ layers were separated by enzymatic treatment. The PS was plated on FIBRONECTIN-coated glass chamber slides. The PS explants (*grey*) attach to the glass and mesenchymal cells migrate outwards to form a mesodermal sheet (*green*) over the course of several days. Direction of migration is indicated by red dotted arrows. Right-to-left movement (*red arrow*) of Venus + cells is indicated as they move away from a *Gata6* ^*H2B-Venus/+*^ PS explant over the course of 3 h. Images were captured every 7 min. Proximal (Pr), distal (D), anterior (A), posterior (P), right (R), left (L), primitive streak (PS), mid streak (MS). Nuclei are stained with Hoechst (*blue*)

*Gata6*^*H2B-Venus/+*^ embryos were morphologically indistinguishable from wild-type littermates except for their fluorescence. In wild-type embryos, GATA6 protein was expressed throughout the extraembryonic VE (exVE) and embryonic VE (emVE) at E5.5. Venus was also detected in the VE at E5.5, and optical sectioning confirmed that the reporter was not active in the Epi or extra-embryonic ectoderm (Fig. 4a). At E6.0, prior to formation of the primitive streak that is (referred to as pre-streak), expression of Venus continued in throughout the VE. Co-expression of GATA6 and Venus was observed in the emVE on the surface of the embryo, but neither were detected in the Epi or extra-embryonic ectoderm (Fig. 4b). However Venus was not activated in all GATA6+ cells of the VE, resulting in a mosaic pattern of Venus expression in the exVE (Fig. 4a) that was also evident at later stages (Figs. 4b, an Fig. 5a). One possible explanation for this observation could be that the *Gata6* locus is subject to mono-allelic expression in certain tissues.

### Activity of *Gata6*^*H2B-Venus*^ during gastrulation

**Fig. 6:**
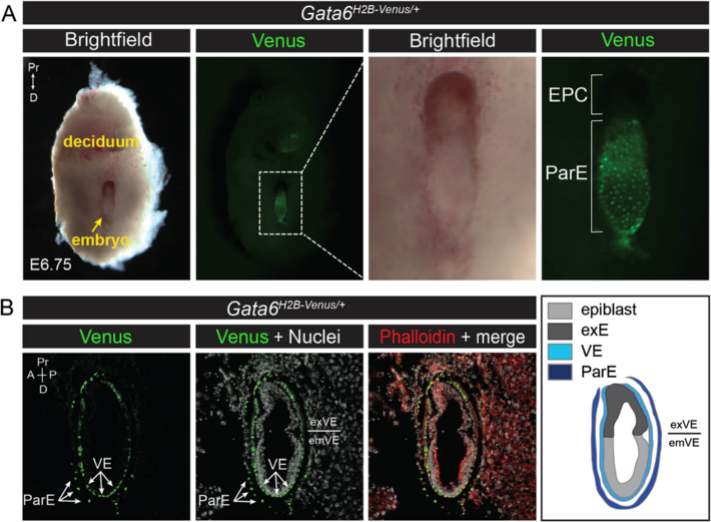
Expression of *Gata6* ^*H2B-Venus*^ reporter in parietal endoderm. **a** Wholemount brightfield and fluorescence images of a bisected deciduum containing an E6.75 *Gata6* ^*H2B-Venus/+*^ embryo. Nuclear localized expression of Venus was observed in cells of the ParE, part of Reichert’s membrane that derives from the PrE lineage. **b** Bissected decidua containing *Gata6* ^*H2B-Venus/+*^ embryos at E6.75 were stained for Phalloidin (*red*) and cryosectioned. Saggital sections show expression of Venus in the ParE and VE. Schematic diagram depicts the layers of Epi (*light grey*), extraembryonic ectoderm (exE, *dark grey*), VE (*light blue*) and ParE (*dark blue*). Ectoplacental cone (EPC), parietal endoderm (ParE), visceral endoderm (VE), extraembryonic VE (exVE), embryonic VE (emVE), proximal (Pr), distal (D), anterior (A), posterior (P)

Next, expression of Venus was characterized during gastrulation stages. Gastrulation begins at E6.25 when Epi cells in the proximal posterior portion of the embryo form the primitive streak (PS). The PS is a region characterized by an epithelial-to-mesenchymal transition whereby ingression of mesoderm and endoderm progenitors results in their migration anterolaterally to populate the space in between two apposed epithelia, the Epi and VE. At the early-streak (ES, E6.75) stage, Venus was observed in the ParE (Reichert’s membrane) upon bisection of decidua that contained *Gata6*^*H2B-Venus/+*^ embryos (Fig. 6a-b). Expression of Venus was mosaic in the VE at E6.75 and mid-streak (MS, E7.0) stages of *Gata6*^*H2B-Venus/+*^ embryos, and did not always correlate with expression of endogenous GATA6 (Fig. 5a). It is possible that expression of *Gata6* mRNA and protein are dynamically changing in the VE at these stages. It is also possible that there are differences in translation regulation of *Gata6* mRNA compared to the reporter mRNA which lacks the wild-type 3′UTR.

Sections through the PS region at E7.0 revealed Venus and GATA6 expression in cells that had left the Epi epithelium (Fig. 5a). These cells may represent the earliest cardiac mesoderm progenitors that will populate the primary and secondary heart fields. To visualize movement of ingressed cells *in vitro*, we performed PS explants that were cultured and time-lapse imaged. The posterior portion of the Epi was dissected from E6.5 embryos, treated with pancreatin/trypsin enzymatic digestion to remove the endoderm and wings of mesoderm, and then cultured on fibronectin-coated chamber slides [55, 56]. Mesoderm precursors migrated away in a centrifugal fashion thus forming what is referred to as a mesodermal sheet that surrounded the original explant. When PS explants were cultured from *Gata6*^*H2B-Venus/+*^ embryos, migrating cells expressed Venus, and individual nuclei could be identified and followed over time (Fig. 5b).

**Fig. 7:**
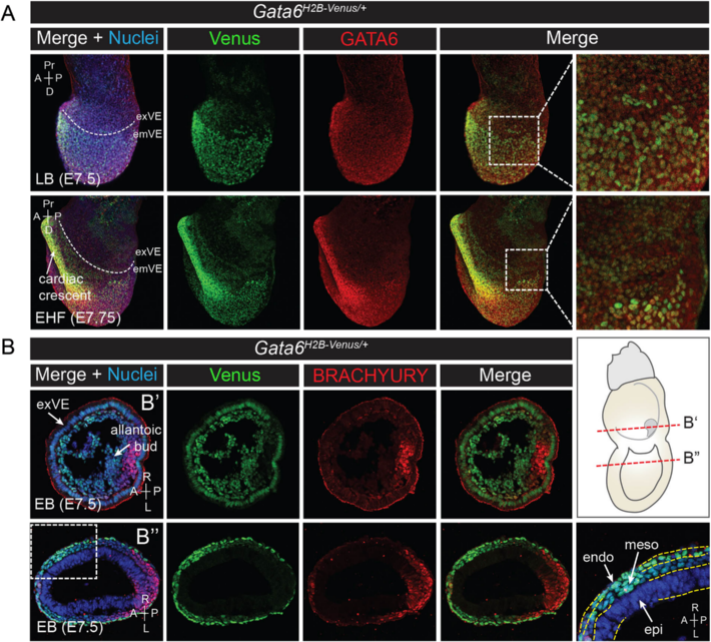
Expression of Venus yellow fluorescence at allantoic bud and headfold stages. **a** Wholemount immunofluorescence for GATA6 on *Gata6* ^*H2B-Venus/+*^ embryos at late bud (LB, E7.5) and early headfold (EHF, E7.75) stages. Venus is expressed in the surface endoderm and headfold. Dashed lines demarcate the border between exVE and emVE. Higher magnification views show low-level mosaic Venus expression in the exVE in the boxed regions. **b** Immunofluorescence for BRACHYURY (*red*) on *Gata6* ^*H2B-Venus/+*^ embryos at early bud (EB, E7.5) stage. Expression of Venus is observed in lateral and anterior mesoderm and endoderm and is distinct from expression of BRACHYURY. Sections through the extraembryonic region (B’, *top row*) and embryonic region (B”, *bottom row*) are indicated by dashed lines on the schematic diagram to the right. A close-up of the region within the dashed white box shows expression of Venus in the emVE and nascent mesoderm (meso), but not in the Epi. Three embryonic tissue layers (emVE, meso, epi) are delineated by dashed yellow lines. Non-nuclear red fluorescent signal that appears apically on the VE is thought to be non-specific binding of the GATA6 antibody to the surface of the extraembyonic tissue

The next stage of development is defined by allantoic bud formation, axial extension from the node, and appearance of headfolds. At the late bud (LB, E7.5) and early headfold (EHF, E7.75) stages, Venus was expressed mosaically in the exVE and throughout the emVE. Venus, together with endogenous GATA6, was strongly expressed in the anterior definitive endoderm, cardiac crescent, and lateral plate mesoderm (Fig. 7a). Venus expression continued in the mesoderm, although it did not appear to coincide with BRACHYURY in the posterior embryonic and extra-embryonic mesoderm at the early bud (EB, E7.5) stage, indicating that Venus was labeling a specific sub-population of mesoderm and/or endoderm progenitors which was distinct from BRACHYURY labeled cells (Fig. 7b). Thus, as cells exit the primitive streak, they likely downregulate BRACHYURY and activate GATA6.

**Fig. 8:**
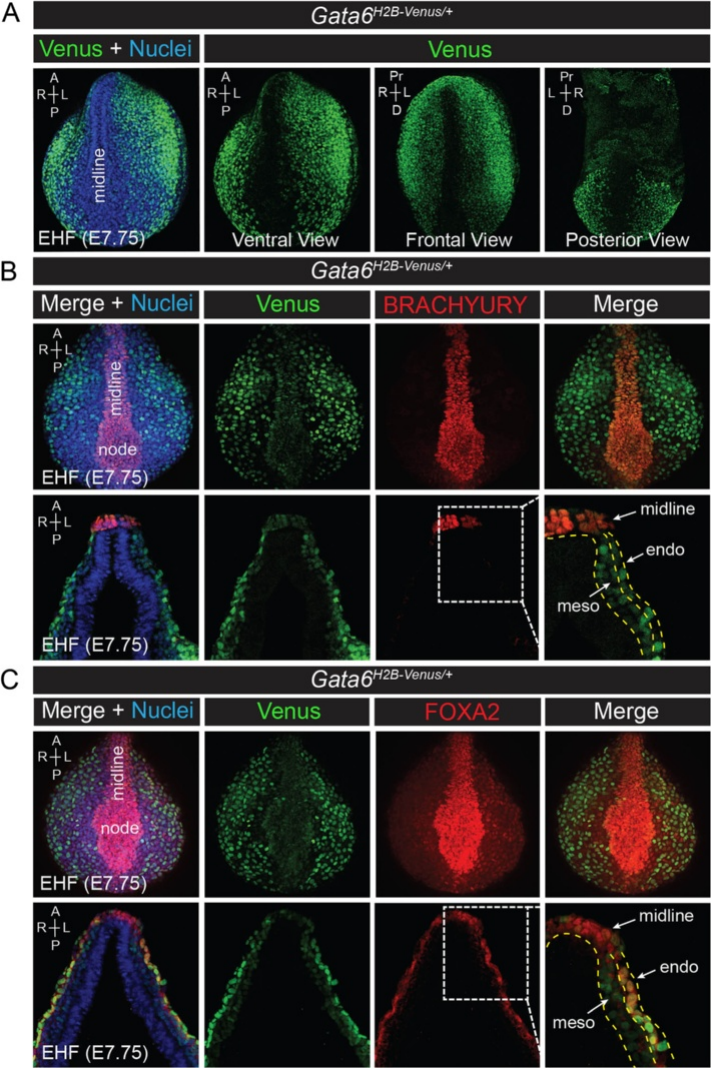
Venus is expressed in the mesoderm and endoderm on the surface of the embryo, but not in the midline. **a** Alternative views (ventral, frontal, posterior) depicting regions of Venus expression (green) in *Gata6* ^*H2B-Venus/+*^ embryos at E7.75. Venus is excluded from the midline. Surface views are renderings of z-series images. Anterior (A), posterior (P), right (R), left (L), proximal (Pr), distal (D). Nuclei are stained with Hoechst (*blue*). **b** Immunostaining for BRACHYURY (*red*) labels the midline in *Gata6* ^*H2B-Venus/+*^ embryos at E7.75. Transverse sections showing mesoderm (meso) and endoderm (endo) layers. **c** Immunostaining for FOXA2 (*red*) shows very high expression in the midline and lower levels of expression in the surface endoderm in *Gata6* ^*H2B-Venus/+*^ embryos at E7.75. Venus (*green*) coincides with FOXA2 in most cells of the surface endoderm, however it is not co-expressed in the midline. Transverse sections showing mesoderm (meso) and endoderm (endo) layers

Exclusion of Venus + endoderm cells from the midline was evident in ventral, frontal, and posterior views of *Gata6*^*H2B-Venus/+*^ embryos at headfold stages (E7.75) (Fig. 8a). This was confirmed by immunostaining for BRACHYURY and FOXA2, two transcription factors that are expressed along the midline at E7.75 [57]. Trace expression of Venus was detectable in the midline at early headfold (EHF) stages, likely due to perdurance. However, expression of Venus mostly did not coincide with that of BRACHYURY or FOXA2 in the midline (Fig. 8b) as well as in the streak (data not shown). At this stage (EHF), Venus was expressed in both the mesenchyme and endoderm. Expression in the endoderm was confirmed by colocalization with FOXA2 in the surface endoderm (Fig. 8c).

### *Gata6*^*H2B-Venus*^ expression in later stage embryos and adults

**Fig. 9:**
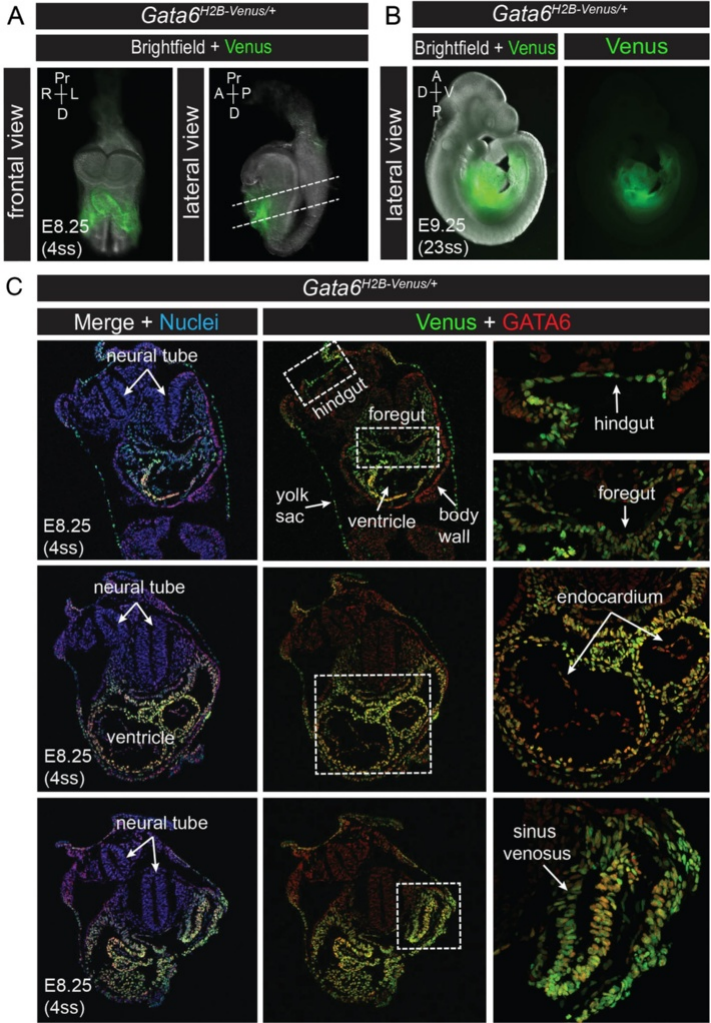
Expression of Venus at somite stages. **a** Frontal and lateral views of wholemount *Gata6* ^*H2B-Venus/+*^ embryos merged with brightfield images at E8.25 (4 somite stage, ss). Venus is expressed in the heart, sinus venosus, and gut endoderm. **b** Lateral views of wholemount Venus expression at E9.5 (23ss) in *Gata6* ^*H2B-Venus/+*^ embryos. **c** Immunofluorescence for GATA6 (*red*) on transverse sections through a *Gata6* ^*H2B-Venus/+*^ embryo. Varying levels of GATA6 co-expression with Venus (*green*) can be seen in the gut endoderm and heart. Some tissues express GATA6 exclusively, for example the body wall and endocardium, while other tissues express Venus only, such as the yolk sac and weak expression in the pharyngeal mesoderm. Nuclei are stained with Hoechst (*blue*)

From E8.25 (4 somite stage, ss) to E9.5 (23ss), wholemount expression of Venus was observed in the heart and gut endoderm (Fig. 9a–b). Sections through *Gata6*^*H2B-Venus/+*^ embryos at E8.25 revealed strong expression of the reporter in all regions of the gut, the heart myocardium, sinus venosus, and yolk sac. Weaker expression of the reporter was observed in the pharyngeal mesenchyme. There were some discrepancies between endogenous GATA6 and Venus reporter expression. For example, the endocardium and the body wall, which derives from the lateral mesoderm, expressed low levels of GATA6, but did not express Venus. Conversely, GATA6 appeared mostly downregulated in the yolk sac, although Venus reporter expression was still robust (Fig. 9c). While this may reflect delays in the activation and/or downregulation of the reporter compared to the endogenous protein, it is also possible that the reporter may be retained more strongly in cells that are not actively dividing. Similarly, faster growing tissues may dilute the reporter more rapidly.

**Fig. 10:**
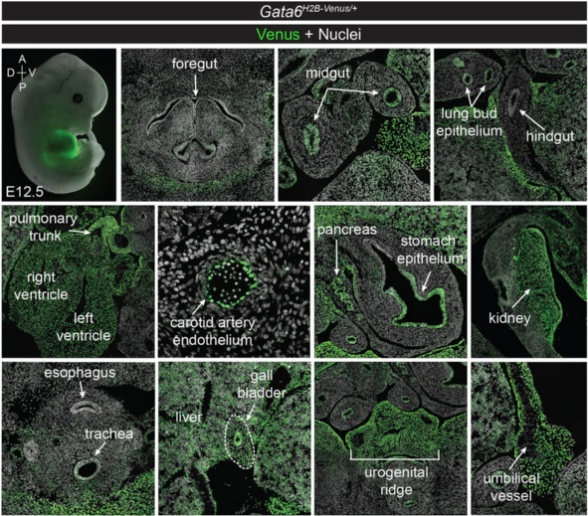
Expression of *Gata6* ^*H2B-Venus*^ reporter at E12.5. Lateral view of a wholemount *Gata6* ^*H2B-Venus/+*^ embryo at E12.5 merged with brightfield (*top left*). Transverse sections at E12.5 showing Venus expression in various endoderm and mesoderm derived organs. Expression is observed in the midgut epithelium, but not the foregut or hindgut. Venus is also seen in the lung bud epithelium, throughout the heart and outflow tract, vascular endothelium, portions of the stomach epithelium, pancreas, kidney, liver, gall bladder, urogenital ridge and umbilical vessels. Venus was not expressed in the esophagus or trachea, Nuclei are stained with Hoechst (*grey*)

By E12.5, expression of Venus was tissue-specific in *Gata6*^*H2B-Venus/+*^ embryos. Low-level expression of Venus was observed in the midgut epithelium whose derivatives express GATA6 later on, but not in the foregut or hindgut. The heart myocardium and outflow tract continued to strongly express Venus. Additional sites of expression detectable at this stage included the stomach epithelium, pancreas, kidneys, lung epithelia, liver, gall bladder, urogenital ridge, arterial endothelium, and umbilical vessels. Consistent with previous characterizations of endogenous GATA6 expression [18], Venus was not observed in certain foregut-derived organs such as the esophagus and trachea (Fig. 10).

**Fig. 11:**
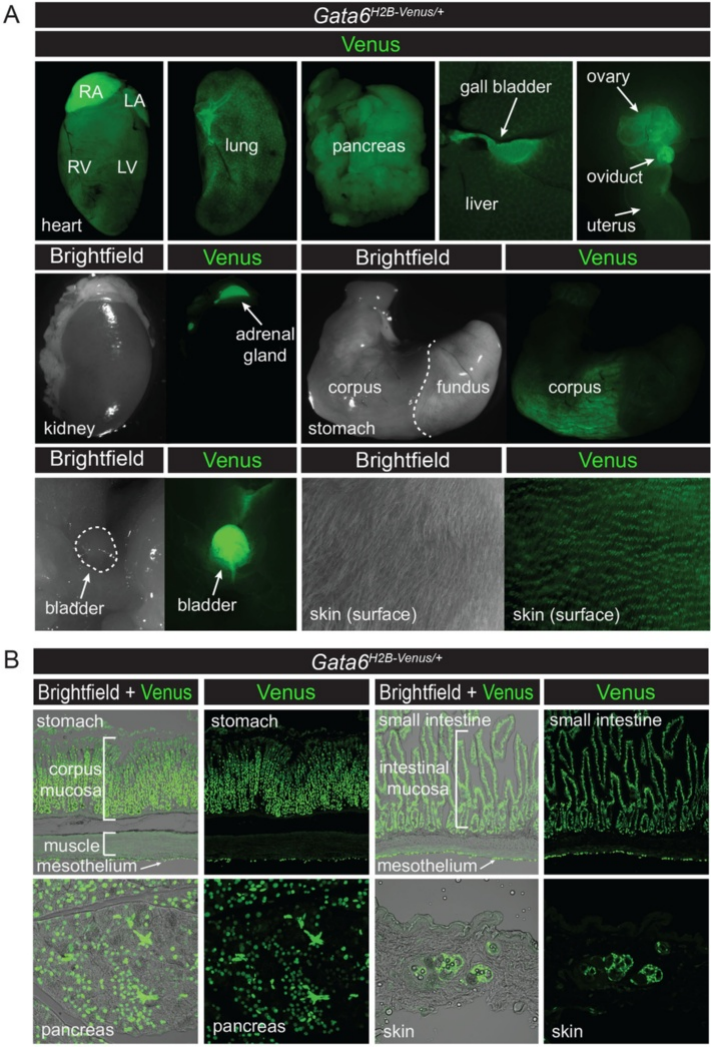
Expression of *Gata6* ^*H2B-Venus*^ reporter in adult organs. **a** In *Gata6* ^*H2B-Venus/+*^ adult mice at 3 months of age, Venus (*green*) is expressed in the heart, lung, pancreas, liver, gall bladder, ovaries, oviducts, uterus, adrenal glands, corpus region of the stomach, bladder, and skin. Right atrium (RA), left atrium (LA), right ventricle (RV), left ventricle (LV). **b** Tissue sections through organs from *Gata6* ^*H2B-Venus/+*^ adult mice at 3 months of age demonstrated expression of Venus in the mucosa and mesothelium of the corpus region of the stomach and small intestine, as well as expression within the pancreas and skin

In *Gata6*^*H2B-Venus/+*^ adults, expression of Venus was observed in organs that have previously been reported to express *Gata6* [18, 32–34, 39]. These include the heart, lung, pancreas, liver, gall bladder, ovaries, adrenal glands, stomach, and bladder. Strong expression of Venus within the skin was also observed (Fig. 11a). Tissue sections of organs from *Gata6*^*H2B-Venus/+*^ mice revealed Venus expression specifically in the mucosa and mesothelium of the corpus region of the stomach and small intestine, as well as expression within the pancreas and skin (Fig. 11b). *Gata6*^*H2B-Venus/+*^ heterozygous adult mice were not recovered at Mendelian ratios (105 mice in total; 44 heterozygous, 61 wild-type), suggesting that adult *Gata6*^*H2B-Venus/+*^ heterozygous mice may have reduced viability.

## Conclusions

Our characterization of the *Gata6*^*H2B-Venus*^ mouse line suggests that it should serve as a useful tool for single-cell resolution imaging of *Gata6* transcriptional activation *in vivo*. Since this is also a loss-of-function allele, it can, in principle, be used in combination with a conditional (floxed) *Gata6* allele to trace the fate of *Gata6* (null) mutant cells after conditional Cre-mediated ablation. Bright, nuclear localization of the Venus reporter allows for direct imaging of the fluorescent protein in fixed tissue samples without the use of an antibody, and also makes this reporter suitable for live imaging and cell tracking.

Co-expression of GATA6 and Venus was well correlated in the embryo from the pre-implantation blastocyst to gastrulation and early somite stages of development. However, some differences were observed between the endogenous GATA6 protein versus the Venus-based transcriptional reporter in terms of levels and localization. For example, in *Gata6*^*H2B-Venus/H2B-Venus*^ homozygous null blastocysts, Venus continued to report transcriptional activity in the absence of GATA6 protein. These data suggest that GATA6 does not feedback on its own transcription, and identify this transcriptional reporter as a readout of *Gata6* expression in the absence of protein function. However, it is also possible that some cells normally express *Gata6* mRNA that is not translated into protein. In the future, this could be determined by quantitative real-time PCR or RNA *in situ* hybridization to detect Gata6 transcripts in specific cells in comparison with GATA6 protein levels. Alternatively, some of these differences may be due to stability of the Venus protein, which may pose challenges for certain experiments. For example, since *Gata6* is activated in all ICM cells prior to the sorting of Epi and PrE lineage progenitors into two distinct layers, then live imaging of the PrE lineage may be difficult if low levels of Venus perdure within Epi progenitors.

In post-implantation embryos, mosaic activation of the *Gata6*^*H2B-Venus*^ reporter in the exVE at E5.5 may potentially reflect mono-allelic regulation of the *Gata6* locus. This characteristic of the reporter could be utilized to investigate dynamics of the expansion of cells in the exVE at early stages of post-implantation development that lead up to gastrulation. During gastrulation, Venus brightly labels both mesoderm and endoderm progenitors and continues to be expressed in some of their derivatives, such as the cells that populate the heart field and gut endoderm. During organogenesis, Venus is expressed in a tissue - specific manner in organ primordia that normally express endogenous GATA6.

In adult mice, the *Gata6*^*H2B-Venus*^ allele may prove useful for studies of disease states, such as cancer models, in which *Gata6* activity may be aberrantly regulated. For example, the reporter may be silenced in tissues that normally express GATA6. Alternatively, the reporter may be ectopically activated and changes in its activity levels may potentially correlate with progression of disease. *Gata6*^*H2B-Venus*^ may also be used as a bright marker of specific cell populations within adult organs, such as in the skin, stomach and small intestine.

Overall, the *Gata6*^*H2B-Venus*^ allele should provide a useful tool for detecting transcriptional activation of the *Gata6* locus that correlates well with endogenous GATA6 protein in both embryonic and adult mice, and can be used for the analysis and isolation of specific cell populations that normally express *Gata6*. Noting the issue of Venus perdurance, it would be of interest to re-target the *Gata6* locus with a destabilized fluorescent protein reporter in order to obtain an improved dynamic readout of *Gata6* transcriptional activity, and not only detect when *Gata6* is activated, but also to determine when the gene is turned off. A destabilized *Gata6* transcriptional reporter might be expected to be dimmer, but accordingly may provide better concordance between *Gata6* gene activity and reporter protein fluorescence. For example, the Venus-NLS-PEST (VNP) fusion protein reporter [58] has a short half-life and has been successfully used to monitor the transcriptional activity of genes, such as *Nanog*, which are dynamically expressed [59]. Similarly, if available, a *Gata6*^*VNP*^ allele could be useful for live quantitative imaging of the rapid changes in *Gata6* expression that occur during various cell lineage specification events involving GATA6.

## Methods

### Mice

To make *Gata6*^*H2B-Venus*^ mice, a targeting vector was constructed by modification of a EUCOMM knockout-first vector [49, 50]. The targeting vector contained an *Engrailed 2* intron and exon with splice acceptor site upstream of the H2B-Venus fluorescent protein reporter [54, 60]. Downstream from this was placed a Neomycin cassette under the control of a human β-actin promoter, which was flanked by *rox* sites for Dre-mediated recombination [61]. Exon 2 was flanked by *loxP* sites, while an *FRT* site positioned upstream of the reporter remained as a remnant of the original EUCOMM knockout-first vector design. The H2B-Venus reporter and Neomycin selection cassette were targeted to Intron 1 of the mouse *Gata6* gene in ES cells of a C57BL6 x 129Sv genetic background. The ES cell line was based on a previously described KH2 clone [52], in which an inducible Gata4-mCherry cDNA had previously been integrated into the Col1a1 locus [50]. Correct targeting to the *Gata6* locus was determined by long-range PCR amplification of the 5’ arm junction using the LongAmp Taq PCR Kit (NEB, #e5200) and the following primers: Gata6_genomic_fwd2: CTTTGAGAGTCTACACCCTTC, R1RN_rev: TGATATCGTGGTATCGTTATGCGCCT (correctly targeted: ~5 kb product). *ColA1*^*TetO-Gata4-mCherry/+*^*;R26*^*M2rtTA/+*^*;Gata6*^*H2B-Venus/+*^ ES cells were cultured under standard ES cell conditions; Knockout DMEM (Life Technologies 10829), 10 % FBS (Hyclone), 1 % L-glutamine (Life Technologies 25030), 1 % non-essential amino acids (Life Technologies 11140), 1 % sodium pyruvate (Life Technologies 11360), 0.1 % β-mercaptoethanol (Life Technologies 21985), 0.01 % LIF (ESGRO, Millipore ESG1107). *ColA1*^*TetO-Gata4-mCherry/+*^*;R26*^*M2rtTA/+*^*;Gata6*^*H2B-Venus/+*^ ES cells from a 129Sv/C57BL6 F1 hybrid (V6.5) background were then injected into C2J blastocysts by the Memorial Sloan Kettering Cancer Center Mouse Genetics Core Facility to generate chimeric mice. All *Gata6*^*H2B-Venus/+*^ mice used in this study contained the Neomycin selection cassette, which has not yet been excised by crossing with Dre-expressing mice [61]. Chimeric males were crossed with CD-1 (Taconic) wild-type females and screened for germline transmission of all three targeted alleles. The *Gata6*^*H2B-Venus*^ targeted allele was bred away from the *ColA1*^*TetO-Gata4-mCherry/+*^ and *R26*^*M2rtTA/+*^ alleles by further crosses with CD-1 wild-type females. Primers for genotyping are as follows: *ColA1*^*TetO-Gata4-mCherry*^, Col1a1_3’_fwd: GCACAGCATTGCGGACATGC, Col1a1_3’_Mutrev: GCAGAAGCGCGCCCGTCTGG, Col1a1_3’_WTrev: CCCTCCATGTGTGACCAAGG (300 bp wild-type band, 500 bp knock-in band); *R26*^*M2rtTA*^, ROSA26_int_for: AAAGTCGCTCTGAGTTGTTAT, rtTA_primer: GCGAAGAGTTTGTCCTCAACC, ROSA26_int_rev: GGAGCGGGAGAAATGGATATG (500 bp wild-type band, 300 bp knock-in band); *Gata6*^*H2B-Venus*^, G6V-5A-Fwd1: CCAGGGAGCTCTGAGAAAAAG, G6V-Rev: CCTTAGTCACCGCCTTCTTG, G6V-wtRev3: GTCAGTGAAGAGCAACAGGT (1 kb wild-type band, 1.2 kb knock-in band). *Gata6*^*H2B-Venus/+*^ mice were maintained on a mixed bred CD-1/129Sv/C57BL6/C2J background in accordance with the guidelines of the Memorial Sloan Kettering Cancer Center Institutional Animal Care and Use Committee. Mice were housed under a 12-h light/dark cycle, and the date of vaginal plug was considered to be embryonic day (E) 0.5. Blastocysts were flushed from oviducts using FHM media (Millipore). The zona pellucida was removed by short incubation in acid Tyrode’s solution (Sigma). Embryos were cultured in KSOM-AA media (Millipore) in a 5 % CO_2_/37 °C atmosphere for time-lapse imaging. Blastocysts were staged according to total cell number and morphology. Post-implantation embryos were dissected in DMEM/F-12 1:1 with 5 % newborn calf serum, and stages were verified using morphological landmarks (E5.5-E7.75) and somite count (E8.25-9.5) [62].

### Embryonic stem cell differentiation

For embryoid body formation, ES cells were resuspended in hanging drops comprising IMDM medium (Life Technologies 12440-His 053) supplemented with 10 % Hyclone FBS, 1 % L-Glutamine, 1 % Penicillin/Streptomycin, 5 % Protein Free Hybridoma Medium II (PFMHII) (Life Technologies 12040–077), 0.5 mM Ascorbic Acid (Sigma A4403), 4.5×10^−4^ M Monothioglycerol (Sigma M6145), and 200 μg/mL Transferrin (Sigma T8158). 1 day later, embryoid bodies were re-plated into non-coated petri dishes and monitored over the course of 3–7 days for fluorescence. For directed differentiation into definitive endoderm using growth factors, cells were incubated in standard ES cell medium (as described above) supplemented with 1 % N-2 (Life Technologies 17502–048), 2 % B-27 (Life Technologies 17504044), and 2.5 μM Y-27632 ROCK inhibitor (Tocris 1254) for 24 h. After 24 h, media was changed to standard ESC media supplemented with 50 ng/mL *E. coli* Activin A (Pepro-Tech, 120-14E) and 5 nM GSK3 inhibitor XV (Calbiochem 361558). After 24 h, media was changed to standard ESC media supplemented with 2 μM Dorsomorphin (Sigma P5499) and 50 ng/mL *E. coli* Activin A (Pepro-Tech, 120-14E), and changed daily for 1–3 days. Transient overexpression of GATA4-mCherry was performed using *Gata6*^*H2B-Venus/+*^*;ColA1*^*TetO-Gata4-mCherry/+*^*;R26*^*M2rtTA/+*^ ES cells [50] incubated with 1 mg/mL Doxycycline (Sigma D9891), replaced daily for 48 h.

### Image acquisition and processing

ES cells and embryoid bodies were imaged on a Zeiss Axio Vert.A1 inverted microscope with a black and white camera (Axiocam MRm). Raw data was processed using Axiovision software and dark field photos were pseudocolored green in Adobe Photoshop. Fixed blastocysts, wholemount embryos (E5.25-7.75), and tissue sections were imaged on a Zeiss LSM880 laser scanning confocal microscope. Blastocysts were imaged along the entire *z*-axis with 1 μm *z*-steps using an EC Plan-Neofluar 40×/1.30 oil immersion objective. For live imaging, blastocysts were imaged with 2 μm *z*-steps and with 15-min intervals. Blastocysts (on glass bottom dishes, MatTek) and PS explants (on 2 chamber coverglass, Lab-Tek) were imaged on the LSM880 inside a heated CO_2_ incubation chamber. Raw data was processed in Zeiss ZEN Black software. Wholemount decidua (E6.5), embryos (E8.25, E9.5, E12.5), and adult organs were imaged on a Leica M165FC dissecting microscope with a color camera (Axiocam MRc) and raw data was processed using Axiovision software.

### Immunofluorescence

Blastocysts were fixed for 10 min in 4 % paraformaldehyde (PFA) at room temperature and immunostained as previously described [63]. Wholemount immunofluorescence (E5.5 to E7.75) was performed on embryos that were fixed in 4 % PFA for 20 min at room temperature followed by 3 washes in PBT (PBS/0.1 % Triton X-100) and stored at 4 °C. Embryos were permeabilized in 0.5 % Triton X-100 in PBS, then blocked in 2 % horse serum in PBT for 45 min at room temperature. Embryos were then incubated overnight in primary antibody diluted in block solution, washed 3×5 min in PBT, incubated with secondary antibody and Hoechst in blocking solution overnight, followed by washing 3×5 min in PBT then storage at 4 °C in PBS. For immunofluorescence on sections, embryoid bodies, halved decidua, and embryos were fixed in 4 % PFA for 2 h (up to E8.25) or overnight (E12.5, adult organs), then washed 3×5 min in PBT, incubated in 30 % sucrose at 4 °C overnight, then embedded in O.C.T. (TissueTek). Cryosections were cut at 12 μm thickness. Tissue sections were washed in PBS, permeabilized for 5 min in 0.5 % PBT, washed in PBS then PBT, blocked in 5 % fetal bovine serum in PBT for 1 h, incubated with primary antibody in PBT overnight, washed 3x5 min in PBT, incubated in secondary antibody and Hoechst overnight, washed 3x5min in PBT, washed in PBS, then coverslipped with Fluoromount G mounting media (Southern Biotech). Primary antibodies used were goat anti-GATA6 (R&D Systems AF1700, 1:100), rabbit anti-NANOG (Cosmo Bio, 1:500), goat anti-BRACHYURY (R&D Systems AF2085, 1:100), and rabbit anti-FOXA2 (Abcam ab40874, 1:1,000). Secondary antibodies used were Alexa Fluor donkey anti-goat 568 (Life Technologies, 1:500) and Alexa Fluor goat anti-rabbit 568 (Life Technologies, 1:500). Fixed nuclei were stained with Hoechst 33342 (Life Technologies, 1:500). Venus fluorescence was imaged directly without the use of antibodies, except for cryosections of adult tissues that were stained with rabbit anti-GFP (Abcam ab290, 1:500) and Alexa Fluor goat anti-rabbit 488 (Life Technologies, 1:500).

### Quantitative analysis of reporter co-localization in pre-implantation stage embryos

Blastocyst images were segmented using the algorithm MINS (Modular Interactive Nuclear Segmentation) for fluorescence quantification (http://katlab-tools.org) [64]. Confocal *z*-stacks were processed with MINS for nuclear segmentation as described [65]. Fluorescence decay along the *z*-axis for each nuclei was corrected using a factor dependent on the position of the nucleus in *z*, as described [65]. PrE and Epi populations were identified manually based on the expression of GATA6 and NANOG, respectively. RStudio was the implementation of R used for all analyses. CSV file containing the raw data for Fig. 2 is provided in the Additional file 1. R-script used for analysis is available upon request. Pearson’s product moment correlation was used to assess the correlation between GATA6 and Venus levels in the PrE at each blastocyst stage. Statistical differences in Venus expression between blastocyst lineages were tested performing analysis of variance (ANOVA) on the average fluorescence level for all cells in each lineage, in each embryo (plotted in Fig. 2c). Tukey’s range test was used as the post-hoc test to determine the groups responsible for the statistical difference.

### Primitive streak explants

The posterior region encompassing the primitive streak (PS) and overlying visceral endoderm was dissected from E6.5 embryos following removal of Reichert’s membrane. The tissue was then incubated for 5–10 min in 2.5 % pancreatin (Sigma) and 0.5 % trypsin (Calbiochem), and washed in PBS. The VE overlying the PS was removed by pipetting, and germ layers were further separated using Tungsten needles to remove the nascent mesodermal wings. PS explants were plated on 2-well chamber coverglass (Lab-Tek) that had been coated with FIBRONECTIN from bovine plasma (SIGMA F1141, 20 μg/mL incubated overnight at 37 °C, washed three times with PBS). Explants were cultured at 37 °C with 5 % CO_2_ in high-glucose DMEM supplemented with 1 % L-Glutamine, 1 % Penicillin/Streptomycin, and 10 % fetal bovine serum. Attachment of explants occurred overnight and growth of the mesodermal sheet occurred over the course of 2–3 days. Time-lapse imaging was performed on the LSM880 confocal laser scanning microscope using a CO_2_ incubation chamber heated to 37 °C.

## Endnote

^1^The *Gata6*^*H2B-Venus/+*^ mouse line described in this report has been submitted for inclusion in The Jackson Laboratory Mouse Repository and will be made available as JAX Stock Number 028096 STOCK Gata6 < tm1(HIST1H2BB/Venus)Hadj>/J.

## Additional file

Additional file 1:
**Figure 2 raw data.** (CSV 163 kb)

## Abbreviations

ICM: Inner cell mass
TE: Trophectoderm
PrE: Primitive endoderm
Epi: Epiblast
ParE: Parietal endoderm
VE: Visceral endoderm
exVE: Extraembryonic VE
emVE: Embryonic VE
exE: Extraembryonic ectoderm
PS: Primitive streak
ES: Early streak
MS: Mid-streak
EB: Early bud
LB: Late bud
EHF: Early headfold
